# Medical Items

**Published:** 1911-03

**Authors:** 


### MEDICAL IETMS

### ANTI-MENINGITIS SERUM.

The Rockefeller Institute for Medical Research, in accordance with an announcement made last summer, now gives notice that it has discontinued the general distribution of anti-meningitis serum which it has undertaken without charge ever since the discovery of this remedy for cerebro-spinal meningitis. The effectiveness of this remedy in that form of meningitis which is caused by the Dipolococcus intracellularis (Weichselbaum) having been generally accepted by medical authorities throughout the world, it has seemed appropriate that The Rockefeller Institute should devote to other lines of investigation the funds hitherto needed for the gratuitious distribution of the serum, handing over to the public health authorities of municipalties and states, and to commercial establishments, the routine preparation of the serum for general use. The anti-meningitis serum will thus take its place with vaccine and diphtheria anti-toxin as an approved agency for the protection of public health.

The Board of Health of the City of New York is the first of American boards of health to undertake the regular production of anti-meningitis serum. It will provide for the free distribution of serum to all hospitals in the city, and, at the outset, to all physicians who apply for it. Later, the gratuitious distribution other than to hospitals will be limited to those cases in which the physician certifies to the hardship that would be caused by a money charge. All others will be required to pay for the serum at a price covering its estimated cost. Pending the production of the serum in other localities, the New York Board of Health will, as a matter of humanity, supply such urgent requests as may come to it from outside the state, but this provision will probably be necessary only a short time. Within the City of New York the Board of Health will designate a few stations where serum will be kept on hand.

The statistics show that the death rate from cerebro-spinal meningitis has been reduced to less than a third of its former amount by the early use of anti-meningitis serum. That statistics may be reliable, however, it is important that all distributing agencies should provide means for controlling the bacteriological diagnosis. Otherwise the serum will undoubtedly be applied in some cases of meningitis due to causes which are not subject to the action of this serum, and not a few cases of epidemic meningitis will be deprived of the benefit of its use.

The serum is administered by being injected into the spinal canal by means of lumbar puncture, an operation which is also required to secure the fluid for bacteriological diagnosis; and several separate injections of the serum are required in treating a given case. The effective employment of the serum is likely, therefore, to be restricted on account of the experience and skill required in its administration and the high cost of the commercial product, unless the preparation, distribution, and, when necessary, administration, are undertaken by state and municipal authorities.

February 13 1911.

JEROME D. GREENE, General Manager.

---

### THE BEST ALKALINE WASH.

By W. HARPUR SLOAN, M. D.

*Chief Ear Department, Medico-Chirugical College, Phila, Penn.*

There are many alkaline preparations on the market that are used daily with varied results in conditions where such a preparation is indicated. I have tried most of them in all conditions, and after an impartial trial, I am compelled to say that the preparation known as "Glyco-Thymoline," by Kress and Owen Co., stands at the head of the list; its formula is one that would commend its use, the ingredients being of an antiseptic and non-irritating nature.

Having formed this opinon of "Glyco-Thymoline," I have concluded to report a few clinical cases where it has given me good results.

Case No. 1.—M. L., age 23 years, came under my care suffering with a distressing case of Ozena. The turibnated bones on both sides of her nose presented a condition of marked atrophy, there was a complete loss of smell and taste, and a formation of crusts in the nasal chamber, the stench of same was foul. She complained of continual headache, and other symptoms of a depleted and run down system. I placed her on a tonic of Iron, Arsenic and Strychnia, internally; locally I ordered the use of "Glyco-Thymoline," in a K. & O. Douche three times a day, diluted. After one month's treatment the crusts had ceased to form, there was a complete restoration of taste and a slight return of smell, the general health was improved, and the patient herself well satisfied with results.

Case No. 2.—C. A., aged 8 years, came to me suffering with a severe Otorrhea following Scarlet Fever. There was a muco-purulent discharge from both ears that rendered the child completely deaf, the auditory canal was excoriated and sore, and the general health below par. I used Cod Liver Oil internally, and syringed the ears three times a day with "Glyco-Thymoline." At the end of one month the discharge of pus had stopped, the hearing much improved, and the child's general health very much better.

### LRYNGEAL OR WINTER COUGHS.

The Journal of Nervous and Mental Diseases in an article by Dr. Walter M. Fleming, says: In acute attacks of laryngeal or winter cough, tickling and irritability of larynx, Antikamnia and Codeine Tablets are exceedingly trustworthy. If the irritation or spasm prevails at night the patient should take one tablet an hour before retiring and repeat it hourly until the irritation is allayed. Allow the tablet to dissolve slowly in the mouth, swallowing the saliva. After taking the second or third tablet the cough is usually under control, at least for that paroxysm and for the night. Should the irritation prevail in the morning or at midday, the same course of administration should be observed until subdued. In neuralgia, in short, for the multitude of nervous ailments, he doubts if there is another remedy so reliable, serviceable and satisfactory, and this, without establishing an exaction, requirement, or habit in the system, as morphine does. —*The New York Medical Journal.*

